# Point-of-Decision Prompts Increase Dietary Fiber Content of Consumers’ Food Choices in an Online Grocery Shopping Simulation

**DOI:** 10.3390/nu12113487

**Published:** 2020-11-13

**Authors:** Kristina Arslain, Christopher R. Gustafson, Devin J. Rose

**Affiliations:** 1 Department of Food Science and Technology, Institute of Agriculture and Natural Resources, University of Nebraska-Lincoln, Lincoln, NE 68588, USA; kristinaarslain@gmail.com; 2 Department of Agricultural Economics, Institute of Agriculture and Natural Resources, University of Nebraska-Lincoln, Lincoln, NE 68583, USA; 3 Department of Agronomy & Horticulture, Institute of Agriculture and Natural Resources, University of Nebraska-Lincoln, Lincoln, NE 68583, USA

**Keywords:** point-of-decision prompt, fiber, diet quality, nutrition information, food choice, online grocery store

## Abstract

Only 5% of Americans consume the recommended amount of dietary fiber. In an online simulated shopping experiment, we examined whether a fiber-focused point-of-decision prompt (PDP) would influence consumers to choose food products that were higher in this important nutrient. We hypothesized that participants exposed to the dietary fiber PDP would choose products with more dietary fiber/serving than those who were not exposed to the PDP. The experiment was completed by 753 participants. Participants were randomly assigned to a condition in which they were not exposed to a PDP (the no-PDP condition), a personalized PDP, or PDP without personalization. Choices in the two PDP conditions were not significantly different. Therefore, the PDP conditions were pooled together into one condition and compared with control participants that did not receive the fiber-focused PDP. Across the three product categories, participants in the PDP condition chose products that had a greater amount of dietary fiber/serving (cereal: 22% increase; bread: 22% increase; crackers: 26% increase; *p* < 0.01) and products that had a greater healthiness rating (cereals (odds ratio (OR): 1.45, 95% confidence interval (95% CI): (1.10, 1.92)), bread (OR: 1.44, 95% CI: (1.09, 1.91)), and crackers (OR: 1.66, 95% CI: (1.25, 2.21)). Overall, the fiber PDP influenced participants to choose healthier products that contained greater amounts of dietary fiber.

## 1. Introduction

Poor diet, which leads to heart disease, certain types of cancer, and type 2 diabetes, is the leading risk factor for premature morbidity and chronic disability in the United States [1,2]. The health care cost of treating these chronic diseases is in excess of USD 50 billion annually [3]. Thus, strategies to promote healthier eating are imperative.

A critical, but under-appreciated, component of a healthy diet is dietary fiber [4]. Dietary fiber is an important nutrient that can aid in weight management, lower blood cholesterol, control blood sugar, maintain regularity, and increase lifespan [5]. Yet currently only 5% of Americans consume the recommended daily intake of fiber (14 g/1000 kcal) [6,7]. Adult women are recommended to consume 25 g of fiber per day [5], but on average consume only 15.5 g dietary fiber/day [8]. Men are recommended to consume 38 g of fiber per day [5], but only consume an average of 18.4 g dietary fiber/day [8].

One way to improve dietary fiber intake is through initiatives implemented in a grocery store or supermarket environment that influence consumers to select higher-fiber products. Many food retailers in the United States and other countries already have resources in the retail environment that can facilitate healthier eating by providing nutrition information that can help consumers identify healthy food products. These resources include nutrition labeling on packaged food products, shelf labeling programs that identify the healthiness of food products (e.g., Guiding Stars in the US; Nutri-score in Europe), health and wellness information on store websites, access to educational grocery tours and registered dietitians, and in-store samples [9].

While these strategies provide the tools to make health judgments among food products, evidence of the effectiveness of these programs suggests that alternative strategies may need to be implemented to effectively promote the purchase of healthier foods. Research that has studied the provision of nutrition information in food retail settings, such as the nutrition facts label or calorie labeling on restaurant menus, finds little to no effect on average in the general population [10,11,12]. Due to the profusion of product options available [13], consumers may limit their attention to a subset of products [14], which may not include the healthiest options, before they begin to consider available nutrition information when making a product choice.

Many existing environmental interventions have been described as point-of-decision prompts (PDPs). PDPs are materials that are available to consumers when they are making a purchasing decision that can influence their choice and include nutrition education programs or point-of-purchase information (i.e., nutrition fact panels and shelf labels) [15,16,17]. More recently, literature has begun to develop around health-focused PDPs that are strategically located to influence the consumer by making health attributes salient before the narrowing of food choices within a category has begun [18]. This literature builds on research on PDPs in the context of promoting physical activity [19,20,21]. The use of strategically placed, simple messaging in the realm of food purchases is a recent approach to promoting healthier food choices. The existing research in this area has found that health-focused point-of-decision messages improved the healthiness of consumer grocery purchases [18,22,23]. A strategy that increases consumer motivation to buy healthy foods at the start of the food decision process may therefore improve the nutritional quality of food choices by influencing the set of products and the nutrition information the shopper considers. Consumers may be more likely to consider health in their shopping choices if they are presented with a health-focused PDP before they begin to determine which products they will consider within a category.

Although previous PDP studies have improved consumer food choice simply by reminding consumers about health and the importance of eating healthy foods [23], a PDP could be designed to target important but under-consumed nutrients. Previous research on the effect of PDPs [18,22] and health primes [23] on food choices in food retail settings have examined messages promoting the consumption of a broad category of food, such as “healthy foods” or “fruits and vegetables.” It is important to understand if a PDP can be used to target a specific under-consumed nutrient while also improving the general healthiness of product choices.

PDPs targeting key nutrients could provide simple educational messages to consumers as part of the prompt. For dietary fiber, in particular, many people are unaware of many of the health benefits of fiber [24]. While the majority of Americans realize that fiber helps with digestion (85%) and weight management (72%), far fewer realize that fiber also helps with heart health (52%) and blood sugar control (43%) [24]. Even fewer Americans likely realize that fiber improves the gut microbiota since this is a new benefit that members of the scientific and health communities are still working to fully document [25]. Consumers may be more motivated to increase their fiber consumption if they understand its health benefits [26].

In addition to the need to further study the use of PDPs in a grocery store environment, there is also a need to study its use in the online grocery shopping environment. Online grocery shopping is growing in popularity [27], but research on the feasibility of online nutrition promotion initiatives is limited [28]. In one poll, fewer consumers indicated looking at nutrition information when shopping for groceries online compared to a physical store [29], suggesting that health promotion interventions may be more critical in the online environment than in physical retail outlets. A health-focused PDP could be easily implemented in an e-commerce food shopping setting to influence consumers to make healthier food selections.

**Figure 1:**
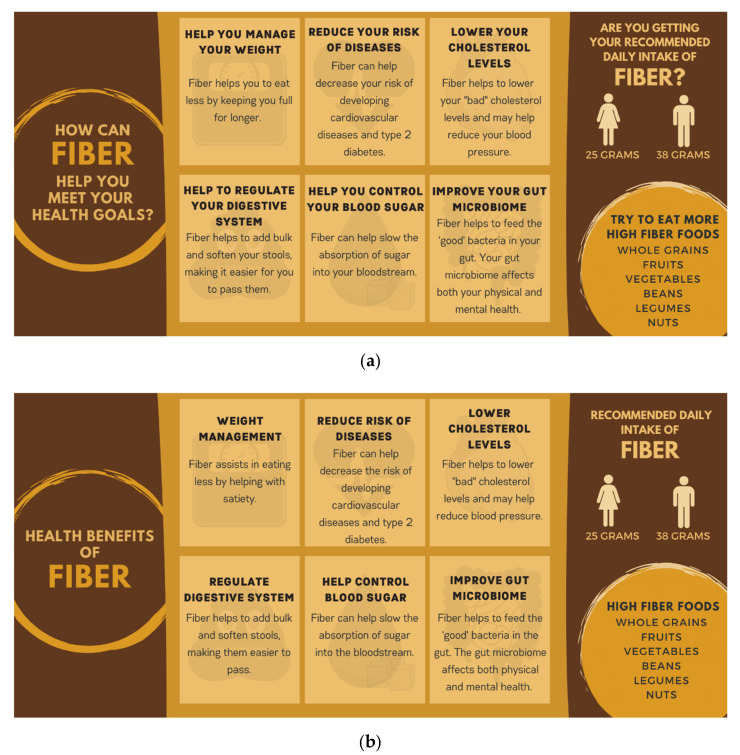
Point-of-decision prompts (PDP) with (**a**) and without (**b**) personalization that participants in the PDP conditions viewed before beginning the shopping task. The authors developed the figures using the graphic design website Canva (www.canva.com) [31].

In this research, we simulate an online shopping experience to examine the effect of a PDP about the health benefits of fiber consumption on fiber content and an overall measure of healthfulness in three grain-based product categories that have significant variation in fiber levels. Our experimental design examined the influence of two slight variants of a core PDP message, compared with a no-PDP condition. The two PDPs contained the same content, but one used personal pronouns (e.g., you, your) in an effort to evoke a more personal connection to the message, while the other did not use pronouns (Figure 1). This was done because the World Health Organization reports creating a personal relevance to an issue can help make health promotion more effective [30]. We found no significant differences in outcomes in response to the two PDPs; therefore, in this paper, we have pooled the data from the two PDP conditions (termed pooled-PDP (P-PDP)) and compare choices in the presence of a PDP with choices made in the no-PDP condition. We hypothesized that participants who were presented with the dietary fiber PDP at the start of the food choice process would choose products with greater fiber content—and healthier products overall—than participants who were not presented with the PDP.

## 2. Materials and Methods

### 2.1. Survey Design

We examined how people’s choices are influenced when they are exposed to a PDP in an online food choice experiment designed to mimic a person’s online grocery shopping experience. The research consisted of two sections: (1) a shopping task in which participants made hypothetical food choices from three product categories: bread, cereal, and crackers; and (2) a survey. The survey included questions about product choices, typical shopping practices, and demographic variables. This survey was created in Qualtrics XM (www.qualtrics.com, 2020, SAP, Provo, UT, USA) and distributed to adults (≥19 years old) in the United States via Amazon Mechanical Turk from 15–20 April 2020. The University of Nebraska-Lincoln Institutional Review Board (IRB) approved the research (IRB protocol #20171017580EX). All participants provided informed consent before participating in the research.

Participants in the research were randomly assigned to a control group (no-PDP) or one of two PDP groups. The two PDP versions contained the same information about the health benefits of fiber consumption but differed slightly in the presentation of the message (Figure 1). One group saw a PDP that was written to evoke a personal connection to the messages by using personal pronouns such as “you” and “your”. We refer to this condition as the personalized PDP. The message in the other group replaced the personal pronouns with impersonal articles. We refer to this condition as the PDP without personalization. Participants in the two PDP groups viewed the PDP just before beginning the shopping task, while control group participants immediately began the shopping task.

**Figure 2:**
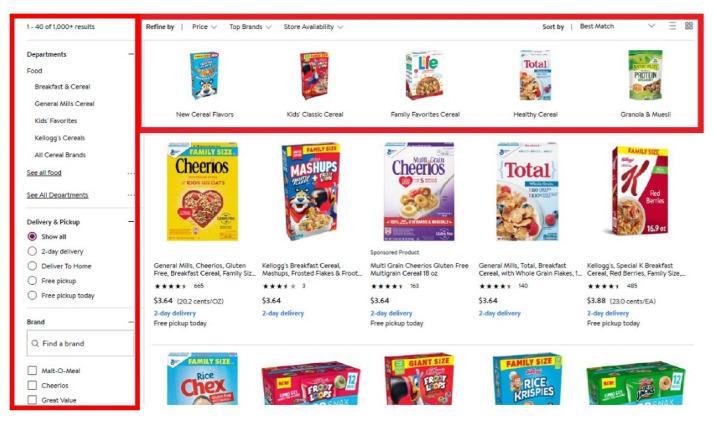
Screenshot of the online grocery shopping experience for a large grocery store chain. As highlighted by the red boxes, shoppers are immediately able to refine the product options and limit the choices they want to consider.

In the shopping task, participants made cereal, bread, and cracker choices. Participants read an introductory text about the shopping task at the start of the survey that told them to imagine they were making real choices with real money, which has been found to reduce biases in hypothetical choices [32]. Before choosing the specific item to purchase, participants decided whether to examine all product options (*n* = 33 for each category: cereal, bread, and crackers) or to view a subset of products (*n* = 11 per subset), reflecting features in many online shopping environments that allow shoppers to easily examine a subset of products (Figure 2). The subsets categorized the products into less healthy, moderately healthy, and healthy options, using a rubric as described in Section 2.2. To avoid prompting participants to think of the subgroups according to the health of the products, the subsets were instead described by the types of products they contained. For example, the cereal sets were labeled as “Cereals such as Frosted Flakes, Froot Loops, Reese’s Puffs”, “Cereals such as Corn Flakes, Crispix, Special K”, “Cereals such as Cheerios, Wheat Chex, Grape Nuts,” and “All options”.

**Figure 3:**
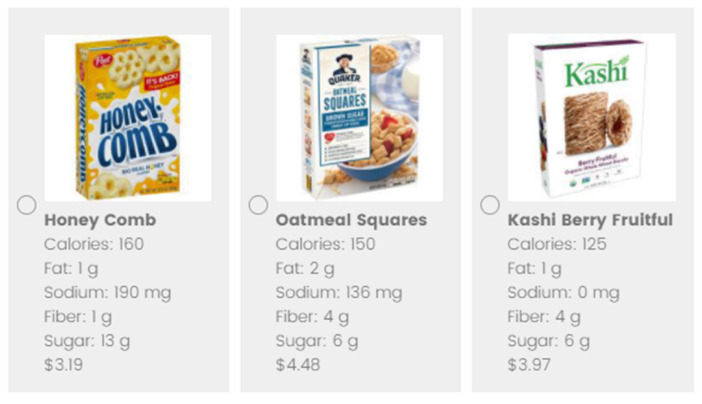
Image of how the cereal choice options appeared to the participant. Under each product option, the calorie, fat, sodium, fiber, and sugar content per serving along with the product price was provided.

After viewing options in each product category, participants were then able to select a product to “purchase”. If a participant did not like any of the products, they had the option to decline to purchase a product for that product category. The no-product option was always listed as the last option, while all other products were presented in random order. The product options were presented in a three-column format with a photograph and the name of each product presented prominently. Underneath each product, the nutrient content per serving for calories, fiber, fat, sodium, and sugar, as well as the price was listed (Figure 3). After making choices in all three product categories, participants answered survey questions about their choices, typical shopping practices, and demographics.

### 2.2. Allocation of Products into Healthiness Categories

We used the Guiding Stars rubric to categorize foods into the three product subsets (less healthy, moderately healthy, and healthy options) (https://www.guidingstars.com). The Guiding Stars system grades the healthiness of products on a 0–11 point scale based on their nutrient content. Products gain points based on meeting certain thresholds for vitamins, minerals, fiber, whole grains, and omega-3 fatty acids, and lose points for surpassing amounts of saturated fat, trans fat, added sodium, added sugar, and artificial colors in a standardized 100 calorie portion. The least healthy products earn 0 points on the scale and receive zero stars, followed by one star (1–2 points), two stars (3–4 points), and finally three stars (5–11 points) for the most healthy products. We created subsets of products that received zero, one, and two or three stars in each product category (Appendix A). The two and three-star rated products were combined to make up the healthy subset because there were not enough three-star rated product options to create a separate category. There were three three-star-rated product options in both the cereal and bread categories and one three-star-rated product in the cracker category. As a reminder, each subset contained 11 products, making 33 product options for each of the three food categories.

### 2.3. Survey Analysis

The data were analyzed using R: The R Project for Statistical Computing [33]. Summary statistics, ordinal regression, linear regression, chi-square tests, and *t*-tests were used to analyze differences in fiber content and the healthiness of product choices (dependent variables) between PDP condition groups (independent variable). We analyzed the outcomes of the PDP separately for cereal, bread, and crackers. We considered *p* < 0.05 to be statistically significant.

We first examined outcomes resulting from each PDP and the no-PDP condition, with the no-PDP condition set as the reference category. These results are reported in Appendix B. When we found no significant differences in outcomes between the two PDP conditions, we pooled data from the two PDP conditions into a single PDP condition (P-PDP) for simplicity of exposition. We report the analysis that compared outcomes in the P-PDP and no-PDP, with the no-PDP condition being the reference group. Participants that indicated they would not choose any of the products in a product category were excluded from the analyses for that specific product category for all analyses (initial disaggregated analyses and pooled analyses).

Summary statistics of the sample came from answers filled out in the survey section of the experiment. Characteristics included in our results are sex, age, household income, education, race, and whether the participant was the primary shopper. Respondents indicated their age in 5 year intervals ranging from “19–24 years old” to “65 and older”. Household income was recorded in categories spanning USD 20,000 intervals, beginning with “Less than USD 20,000” and stopping at “USD 100,000 or more.” Respondents selected their highest level of education completed from the options: “Less than high school”; “High school/General Education Development (GED)“; “Some college/associate degree”; “Bachelor’s degree”; “Advanced degree (Master of Business Administration (M.B.A.), Doctor of Medicine (M.D.), Juris Doctor (J.D.), Master of Science (M.S.), Master of Arts (M.A.), Doctor of Philosophy (Ph.D.))”; and “Prefer not to answer.” Participants were asked to select all race options that applied to them. Options included “White,” “Hispanic or Latino,” “Black or African American,” “Native American or American Indian,” “Asian or Pacific Islander”, “Other”, or “Prefer not to answer.” Participants who selected more than one race were then merged into a “Two or more races” category. Participants were asked to answer “Yes,” “Equally shared,” or “No” to the question “Are you the main grocery shopper for your household?”

We examined nutrition outcomes in three ways:

1. We determined the influence of the PDP on the fiber content of participants’ food choices. We calculated the mean fiber content per serving of product choices; 95% confidence intervals and *t*-tests were used to determine significance in mean difference between PDP conditions.
2. We then investigated the influence of the PDP on other nutrients (calories, fat, sodium, and sugar) in the same way we analyzed the influence of the PDP on fiber. We also calculated correlations between fiber content and the other nutrients.
3. We analyzed the influence of the PDP on the healthiness of product choices. The healthiness of products was captured by its Guiding Star rating, which ranged from zero to three stars. The Guiding Stars rating of choices was used as the dependent variable in an ordinal logistic regression model.

In the models reported in the body of the article, we have not included demographic variables. Since participants were randomized into conditions, demographic variables should not affect the impact of the PDP. As a robustness check, we conducted all analyses with the demographic variables included. The inclusion of demographic variables did not affect the estimated impact of the PDP but did require more participants to be dropped from the data set because of “prefer not to answer” responses. We chose to report the version without the demographic variables for simplicity and to avoid removing additional participants from the cereal, bread, and cracker models. The regression results with demographics included are provided in Appendix C.

## 3. Results

### 3.1. Participant Demographics

In total, 753 participants completed the experiment. There were 253 participants in the no-PDP condition, 251 in the personalized PDP condition, and 249 participants in the PDP condition without personalization (and therefore 500 in the P-PDP condition). No significant differences in demographic variables existed between no-PDP condition and the PDP conditions (Table 1). Of the 753 participants, 35.6% of the participants were female, 63.6% were male, and 0.8% preferred not to respond. Most of the participants were within the age range of 25–34 years (47.4% of the sample population) or 35–44 years (25.8% of the sample population). A majority of respondents were the primary household shopper or shared this responsibility with other(s) in the household (>95%), regardless of PDP condition.

### 3.2. PDP Effect on Fiber Content of Choices

**Figure 4:**
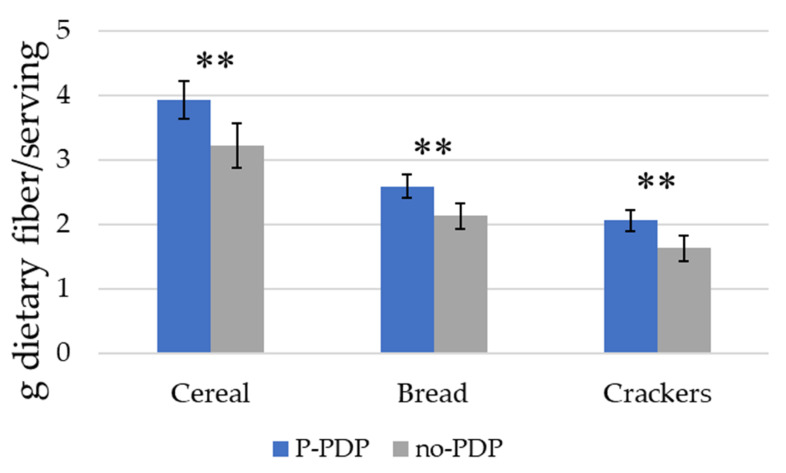
The dietary fiber content of product choices made by participants in P-PDP and no-PDP conditions. Error bars represent the 95% confidence interval around the mean. The analyses included only those subjects that made a product choice; 24 (3.2%), 19 (2.5%), and 18 (2.4%) participants who had selected “none of these” in the cereal, bread, and cracker models were omitted prior to the analyses. This left *n* = 729 cereal choices, *n* = 734 bread choices, and *n* = 735 cracker choices. ** *p* < 0.01. Notes: no-PDP = participants were not exposed to a prompt); P-PDP = pooled prompt condition.

We first examined how the PDP influenced the fiber content among participants’ cereal, bread, and cracker choices. In all product categories, the participants in the PDP condition selected products with significantly more fiber per serving (Figure 4). For cereals, subjects that viewed the PDP before making their product choice selected products with 0.71 g more dietary fiber/serving (*p* = 0.002) than those in the no-PDP condition (a 22% increase). For the bread category, participants in the P-PDP condition chose bread products with an average of 0.46 g more dietary fiber/serving (*p* = 0.001) than those in the no-PDP condition (a 22% increase). Crackers chosen by participants in the P-PDP condition had an average of 0.43 g more dietary fiber/serving (*p* = 0.002) than those in the no-PDP condition (a 26% increase).

### 3.3. PDP Effect on Other Nutrient Content of Choices

**Figure 5:**
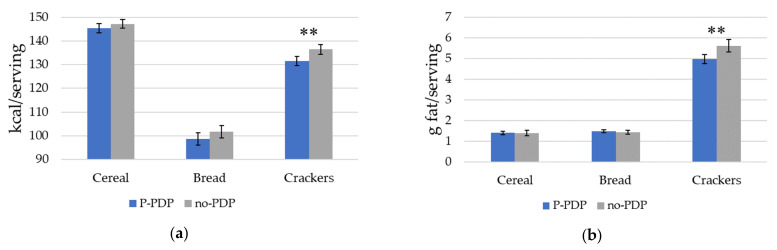

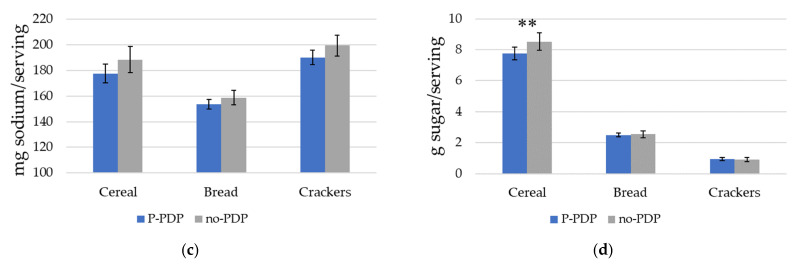
Mean nutrient content of products by participants in the P-PDP and no-PDP conditions. (**a**) Calories; (**b**) fat; (**c**) sodium; (**d**) sugar. Error bars represent the 95% confidence interval around the mean; *p*-values compare significant differences between the mean nutrition content of product choices made by the P-PDP and the no-PDP condition (*t*-test). ** *p* < 0.01.

We next examined the average calorie, fat, sodium, and sugar content of products chosen in the P-PDP and no-PDP groups (Figure 5). For cereals, subjects who viewed the PDP before making their product choice selected products with 0.76 g less sugar/serving (*p* = 0.033) than those in the no-PDP condition (a 9% decrease). For the cracker category, participants in the P-PDP condition chose crackers with an average of 4.95 fewer calories/serving (*p* = 0.002) than those in the no-PDP condition (a 4% decrease). Crackers chosen by participants in the P-PDP condition had an average of 0.63 g less fat/serving (*p* = 0.001) than those in the no-PDP condition (an 11% decrease).

After determining the nutrient differences between products chosen by participants in the P-PDP condition and the no-PDP condition, we calculated correlations between dietary fiber content and the other nutrients in all products included in the research (Table 2). The dietary fiber content per serving in cereals was negatively correlated with the calorie, sodium, and sugar contents. The dietary fiber content in crackers was negatively correlated with all nutrients investigated.

### 3.4. PDP Effect on Healthiness of Choices

**Figure 6:**
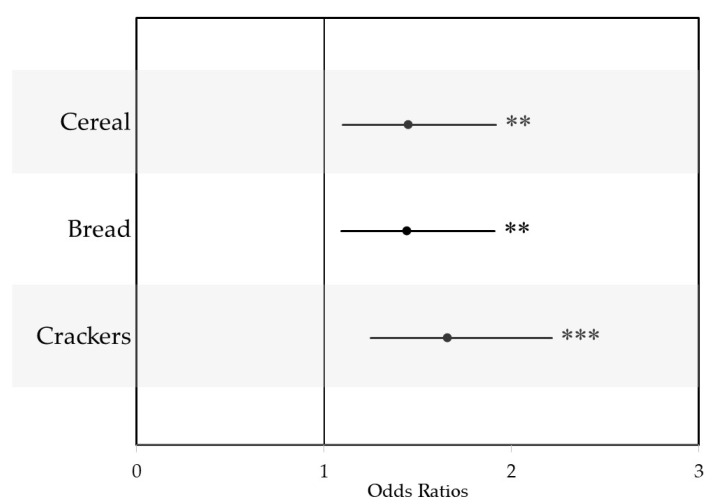
Odds ratios for the influence of a PDP on the healthiness of choices, represented by a product’s Guiding Stars rating with 95% confidence intervals. ** *p* < 0.01, *** *p* < 0.001.

Next, we examined how exposure to the PDP influenced the overall healthiness of participants’ product choices for cereal, bread, and crackers to determine how a fiber-focused PDP impacted the general nutritional quality of food choices. Odds ratios were used to report the effect of the P-PDP on the Guiding Star rating of products selected (Figure 6). Exposure to the PDP led participants to choose a product with a higher Guiding Star rating for cereals (odds ratio (OR): 1.45, 95% CI: 1.10, 1.92), bread (OR: 1.44, 95% CI: 1.09, 1.91), and crackers (OR: 1.66, 95% CI: 1.25, 2.21).

## 4. Discussion

Our research corroborates earlier findings that PDPs increase the nutritional quality of food choices [18], but we additionally find that a PDP highlighting the benefits of dietary fiber-an under-consumed nutrient—improves the healthiness of cereal, bread, and cracker choices in a grocery store environment by increasing the amount of dietary fiber/serving in products chosen by participants in the prompt condition. In all three product categories, participants in the P-PDP condition chose products that contained higher fiber density, but were also more likely to select products with higher Guiding Star ratings, suggesting that, in addition to successfully targeting fiber content, the prompt increased the overall nutritional quality of choices compared to choices made by participants in the no-PDP condition. Our correlation analysis suggests that the higher nutritional quality is due both to higher dietary fiber, which contributes to nutritional quality, but also to correlations between fiber and other nutritional attributes that improve the overall nutritional profile of the foods.

The PDP led to fiber increases of 0.43–0.71 g dietary fiber/serving in each of the three product categories, resulting in a total difference of approximately 1.5 g of dietary fiber/serving across the three food categories. Given that the average fiber intake in the US is 16.2 g/day [8], consumption of one serving of products from each of these categories per day would lead to a 10% increase in total fiber consumption. Currently, the majority of consumer grain-based fiber consumption comes from the high consumption of low fiber foods, so successfully encouraging consumers to choose products with higher fiber content could yield marked increases in consumption of dietary fiber. Participants in the P-PDP condition also selected generally healthier products, as represented by the Guiding Star rating of the products chosen.

Inattentiveness to long-term goals, such as health during decision-making, has been documented in extensive literature on executive function [34,35]. Low executive function is associated with the inability to successfully ignore short-term temptations—such as taste—over long-term rewards that have less of an immediate benefit—such as health [34]. PDPs may work better than nutrition information at reminding consumers of goals for their long-term health. Laboratory studies that simultaneously capture behavioral and neurocognitive data provide evidence on how PDPs may remind one of their long-term health goals. People experience different neural activations when prompted to think about health before making food choices compared to when they are prompted to think of taste or not prompted at all [18]. The neural activation of health-primed individuals resembled dieters who successfully exerted self-control during food choice in an earlier study [36]. Behaviorally, health-primed individuals placed greater value on health attributes and, as a result, were more likely to choose a healthy item [37]. PDPs may be able to help consumers incorporate their long-term goals to balance short-term rewards by recruiting neural systems that are necessary for self-control. Future research should investigate mechanisms by which the PDP affects decision making.

It is important to remind consumers of long-term health goals at the start of their food decision process. With so many product choices within a category, consumers may not consider nutrition information available for products until they have already narrowed their choices down to a subset of choices [14]. Once consumers begin to compare their subset of choices, they may get distracted from the nutrition information by the visuals of the packages [38]. Health-focused PDPs may disrupt both of these processes, leading consumers to consider a broader array of products and reminding them to consider nutrition information. Reminding consumers at the start of their food choice process may better encourage shoppers to consider nutrition in their product selection.

Simulating the shopping experience online directly relates our findings to online grocery shopping experiences. Our research is particularly relevant for the growing segment of the population purchasing groceries online or through an app. An increasing number of consumers have begun to do their grocery shopping online. As of 2019, over 36% of Americans reported that they purchased groceries online [27], and more people have reported using online grocery shopping during the COVID-19 pandemic [39]. Before the epidemic, the Food Marketing Institute and Nielsen predicted that nearly three-fourths of consumers would purchase groceries online by 2024 [40]. With the transition from brick-and-mortar to e-commerce grocery shopping, policymakers, researchers, and public health professionals must understand how to encourage healthier food choices in an online grocery shopping platform. The findings in our study support presenting a health PDP on the computer screen before consumers begin to fill their online grocery cart. However, we view our results as being relevant beyond the online shopping environment. Previous research on PDPs and primes was conducted in retail food stores [6,9], and many consumers continue to predominantly purchase food in-store. While previous PDPs have been physically displayed in-store [6], the rise of “smart” devices that are always with consumers, such as phones and watches, present additional—and customizable—opportunities to prompt consumers when they are in the retail food outlet.

An interesting note about our findings is that the data were collected during the midst of the COVID-19 pandemic. The COVID-19 pandemic has led to increased stress in individuals and an economic downturn. During this time, a third of Americans reported experiencing a high level of psychological distress [41] and purchases of less healthy foods increased [42]. Historically, people consume more unhealthy foods when under these types of pressures [42]. Despite consumers likely feeling stressed, our study still found that the PDP encouraged healthier choices. Our PDP might have been more influential if the data had not been collected during the COVID-19 pandemic. In future work, it may be interesting to collect data using this survey design once the COVID-19 pandemic has passed. This would allow us to examine changes in the effect of the PDP when consumers experience higher than average levels of stress.

A limitation of this study is that product choices were hypothetical. By making hypothetical choices, participants may not have invested as much effort in considering product options or reading product nutrition information. We attempted to address this potential bias by prompting participants to make choices as though they would be actually buying the products, and facing the same budget constraints they do in real life, which has been found to reduce hypothetical bias [32]. While we do not report these data in the paper, analyses show that participants responded to prices in our experiment and were less likely to select higher priced items, suggesting that we were able to mitigate biases related to hypothetical choices.

In conclusion, our data show that participants who were presented a PDP about the health benefits of fiber made healthier product choices within the cereal, bread, and cracker categories by selecting products with higher fiber. This study adds to the burgeoning literature about the use of health PDPs to promote healthy food choices.

## Figures and Tables

**Table 1:** Characteristics of the sample population ^1^.

	Total	no-PDP	P-PDP
Variable	(*n* = 753)	(*n* = 253)	(*n* = 500)
	Count	%	%
Sex			
Female	268	36.0	35.4
Male	479	63.6	63.6
Prefer not to answer	6	0.4	1.0
Age			
19–24	40	4.7	5.6
25–34	357	47.0	47.6
35–44	194	25.3	26.0
45–54	103	14.2	13.4
55–64	43	7.9	4.6
65 and older	12	0.8	2.0
Prefer not to answer	4	0.0	0.8
Household Income			
Less than USD 20,000	54	7.1	7.2
USD 20,000–39,999	146	17.4	20.4
USD 40,000–59,999	177	24.1	23.2
USD 60,000–79,999	179	23.3	24.0
USD 80,000–99,999	100	14.6	12.6
USD 100,000 or more	87	12.6	11.0
Prefer not to answer	10	0.8	1.6
Education			
Less than high school	2	0.4	0.2
High school/G.E.D.	80	10.3	10.8
Associate’s degree or some college	124	18.6	15.4
Bachelor’s degree	400	49.0	55.2
Advanced degree (master’s level or higher)	143	21.7	17.6
Prefer not to answer	4	0.0	0.8
Race			
White	528	71.9	69.2
Hispanic or Latino	37	4.3	5.2
Black or African American	104	14.6	13.4
Native American or American Indian	5	0.4	0.8
Asian or Pacific Islander	35	5.1	4.4
Other	1	0.0	0.2
Two or more	38	3.6	5.8
Prefer not to answer	5	0	1.0
Primary Shopper			
Yes	514	67.6	68.6
Equally shared	207	28.5	27.0
No	32	4.0	4.4

^1^ There were no significant differences between conditions (chi-squared test). Notes: no-PDP = participants were not exposed to a prompt; P-PDP = pooled prompt condition; G.E.D. = General Education Development.

**Table 2:** Correlations between the fiber content and the content of other nutrients per serving in the cereal, bread, and cracker products included in the research (*n* = 33 per category).

Category	Calories	Fat	Sodium	Sugar
Cereal	−0.61 ***	0.09	−0.39 *	−0.49 **
Bread	0.08	0.16	0.06	0.29
Crackers	−0.88 ***	−0.70 ***	−0.55 ***	−0.40 *

* *p* < 0.05, ** *p* < 0.01, *** *p* < 0.001.

## Appendix A

**Table A1:** Nutrition content of product choices within Guiding Star ratings ^1^.

	*n*	Fiber			Calories			Fat			Sodium			Sugar		
		(g/Serving)			(kcal/Serving)			(g/Serving)			(mg/Serving)			(g/Serving)		
		Mean	Min	Max	Mean	Min	Max	Mean	Min	Max	Mean	Min	Max	Mean	Min	Max
Cereal																
0 Stars	11	1.4	0	4	154	140	170	1.8	0	4.5	204	140	270	13.2	12	16
1 Stars	11	2.6	0	4	152	140	160	1.1	0	2.5	241	136	300	8.9	4	12
2 Stars	8	5.3	4	12	145	120	200	1.4	0.5	2.5	137	0	267	6.8	2	13
3 Stars	3	8.0	5	14	123	90	140	1.2	1	1.5	111	0	193	1.0	0	3
Bread																
0 Stars	11	1.0	0.5	2	121	70	160	1.5	0.5	5	196	120	295	3.0	0	5.5
1 Stars	11	1.9	0	3.5	79	45	125	1.1	0.5	2	126	85	180	1.5	0.5	3.5
2 Stars	8	3.9	1.5	8	92	60	120	1.8	1	3	153	115	220	2.8	0.5	5
3 Stars	3	3.7	3	4	93	50	130	2.0	1	2.5	117	85	135	1.5	0.5	3
Crackers																
0 Stars	11	0.4	0	1	147	140	150	7.3	5	9.5	259	210	380	1.5	0	4
1 Stars	11	1.8	0	3	132	120	140	3.9	1.5	5.5	147	85	250	1.1	0	3
2 Stars	10	3.5	1	7	121	69	150	4.0	0	8	172	115	270	0.1	0	0.5
3 Stars	1	7.0	7	7	67	67	67	0.0	0	0	117	117	117	0.0	0	0

^1^ 0 stars = least healthy; 1 star = moderately healthy; 2 or 3 stars = most healthy.

## Appendix B

**Table A2:** Estimate and 95% confidence interval around the estimate for nutrient content of products by participants in the personalized PDP and PDP without personalization compared with the no-PDP condition ^1^.

Nutrients	Personalized PDP				PDP without Personalization			
	Estimate	CI Low	CI High		Estimate	CI Low	CI High	
Fiber								
Cereal	0.64	0.10	1.19	*	0.78	0.23	1.33	**
Bread	0.54	0.20	0.88	**	0.37	0.03	0.71	*
Crackers	0.39	0.07	0.72	*	0.47	0.14	0.79	**
Calories								
Cereal	−0.34	−3.83	3.15		−3.28	−6.78	0.22	
Bread	−2.55	−7.74	2.64		−3.57	−8.81	1.68	
Crackers	−4.65	−8.48	−0.81	*	−5.25	−9.07	−1.42	**
Fat								
Cereal	0.03	−0.15	0.21		0.00	−0.18	0.18	
Bread	0.02	−0.13	0.16		0.11	−0.03	0.26	
Crackers	−0.56	−1.01	−0.12	*	−0.70	−1.14	−0.26	**
Sodium								
Cereal	0.64	0.10	1.19	*	0.78	0.23	1.33	**
Bread	−5.27	−12.76	2.23		−5.10	−12.67	2.47	
Crackers	−9.66	−20.84	1.52		−8.99	−20.13	2.16	
Sugar								
Cereal	−0.89	−1.70	−0.07	*	−0.64	−1.46	0.17	
Bread	0.06	−0.20	0.31		−0.13	−0.39	0.13	
Crackers	0.00	−0.20	0.21		0.00	−0.20	0.21	

^1^ Estimate results from linear regression models comparing nutrient content of choices between the personalized PDP, no personalization PDP, and no-PDP conditions. The no-PDP condition was used as the reference group. There were 24, 19, and 18 participants removed from the cereal, bread, and cracker models, respectively. * *p* < 0.05, ** *p* < 0.01.

**Table A3:** Odds ratios (OR) and 95% confidence intervals (CI) for the influence of the personalized PDP, PDP without personalization, and no-PDP on the healthiness of participants’ choices ^1^.

	Personalized PDP				PDP w/o Personalization			
Category	Estimate	CI Low	CI High		Estimate	CI Low	CI High	
Cereal	1.46	1.07	2.01	*	1.44	1.04	1.99	*
Bread	1.44	1.05	1.97	*	1.45	1.05	2.02	*
Crackers	1.52	1.10	2.12	*	1.81	1.30	2.51	***

^1^ Healthiness measured in terms of Guiding Star rating. The no-PDP condition was set as the reference category. There were 24, 19, and 18 participants removed from the cereal, bread, and cracker models, respectively. * *p* < 0.05, *** *p* < 0.001.

**Table A4:** Mean and 95% confidence interval around the mean for nutrient content of products by participants in the Personalized PDP and PDP without personalization conditions ^1^.

	Personalized PDP			PDP w/o Personalization			
Nutrients	Average	CI Low	CI High	Average	CI Low	CI High	*p*-Value
Fiber (g/serving)							
Cereal	3.86	3.48	4.25	4.00	3.70	4.29	0.641
Bread	2.67	2.40	2.94	2.50	2.33	2.67	0.351
Crackers	2.02	1.77	2.26	2.09	1.93	2.26	0.657
Calories (kcal/serving)							
Cereal	146.91	144.20	149.62	143.97	142.14	145.80	0.127
Bread	99.20	95.65	102.74	98.18	95.54	100.82	0.699
Crackers	131.86	128.97	134.76	131.27	129.31	133.22	0.771
Fat (g/serving)							
Cereal	1.42	1.30	1.55	1.40	1.31	1.48	0.775
Bread	1.45	1.35	1.55	1.55	1.47	1.62	0.205
Crackers	5.04	4.73	5.36	4.91	4.69	5.13	0.552
Sodium (mg/serving)							
Cereal	180.42	170.43	190.40	174.75	167.05	182.44	0.455
Bread	153.53	148.50	158.57	153.70	149.88	157.52	0.964
Crackers	189.78	182.06	197.50	190.46	184.91	196.01	0.905
Sugar (g/serving)							
Cereal	7.65	7.09	8.21	7.89	7.47	8.31	0.562
Bread	2.60	2.41	2.78	2.41	2.27	2.54	0.157
Crackers	0.93	0.78	1.08	0.93	0.82	1.03	0.991

^1^*p*-values compare significant differences between the mean nutrition content of product choices made by the Personalized PDP, PDP without personalization condition (*t*-test).

**Table A5:** Odds ratios (OR) and 95% confidence intervals (CI) for the influence of a Personalized PDP (vs. PDP without Personalization) on the healthiness of participants’ choices ^1^.

	Personalized PDP (vs. PDP w/o Personalization)		
Category	Odds Ratio (OR)	CI Low	CI High
Cereal	1.02	0.74	1.40
Bread	0.99	0.72	1.36
Crackers	0.84	0.61	1.17

^1^ Healthiness measured in terms of Guiding Star rating. There were 18, 12, and 13 participants removed from the cereal, bread, and cracker models, respectively.

## Appendix C

**Table A6:** Odds ratios (OR) and 95% confidence intervals (CI) for the influence of the PDP on the healthiness of participants’ choices with demographics ^1^.

	P-PDP (vs. no-PDP)			
Category	OR	CI Low	CI High	
Cereal	1.49	1.13	1.98	**
Bread	1.50	1.13	1.99	**
Crackers	1.72	1.28	2.30	***

^1^ Healthiness measured in terms of Guiding Star rating. Demographic variables (sex, age, years of education, and household income) were included in the regression model. There were 34, 28, and 28 participants removed from the cereal, bread, and cracker models, respectively. ** *p* < 0.01, *** *p* < 0.001.
